# The Stealth and Potentially Fatal Nature of *Kingella kingae* Outbreaks in Daycare Facilities

**DOI:** 10.1093/ofid/ofaf066

**Published:** 2025-02-04

**Authors:** Pablo Yagupsky

**Affiliations:** Clinical Microbiology Laboratory, Soroka University Medical Center, Ben-Gurion University of the Negev, Beer-Sheva, Israel

**Keywords:** children, daycare centers, invasive infections, *Kingella kingae*, outbreaks

## Abstract

Although *Kingella kingae* infections are usually sporadic, outbreaks of *K. kingae* disease have been reported. Outbreaks of invasive *K. kingae* infections in daycare centers were searched through the Pubmed database. Twenty-seven outbreaks have been detected in North America, Western Europe, and Israel. The median age of the 72 affected attendees was 14 months, and the attack rate was 18%. Osteoarthritis was diagnosed in 66 (92%) attendees, and endocarditis in 3 (4%), 2 of whom died. A high prevalence of the invasive strains was found among asymptomatic classmates. Genomic analysis of the available strains identified the highly invasive sequence-type complexes 23/25, 14, or 6 in 12 of 13 (92%) outbreaks. *Kingella kingae* strains causing daycare outbreaks exhibit enhanced colonization, transmissibility, and virulence. Increased awareness of this emerging public health problem and the use of molecular diagnostic methods are recommended for early identification of outbreaks and prevention of fatal outcomes.

## Teaser

Although mild osteoarthritis represented the majority of infections in Kingella kingae daycare center outbreaks, three cases of endocarditis were identified during the outbreaks, two of which were fatal. Increased awareness and the use of molecular tests are needed to identify outbreaks and prevent fatal outcomes.

The growing use of improved culture methods and nucleic acid amplification tests (NAATs) has resulted in the appreciation of *Kingella kingae*'s role as an important pediatric pathogen and the prime etiology of joint and bone infections in children aged 6–48 months [1–3]. The bacterium colonizes the oropharyngeal epithelium after 6 months of age. Its prevalence gradually increases, reaching a 10% to 12% rate during the second year of life, and it declines in older children [4]. The pharyngeal carriage of *K. kingae* is a dynamic event, and new strains are acquired over time, displacing previously colonizing organisms [1, 5]. *Kingella kingae*'s colonization is asymptomatic, playing the dual role of being the initial phase of invasive infections and the source of the person-to-person spread of the bacterium in the community [4–6]. Dissemination of *K. kingae* is facilitated in daycare centers (DCCs) attended by susceptible young attendees [1, 7].

*Kingella kingae* strains carried in the pharynx show variable degrees of invasiveness. Many colonizing strains are uncommon agents of clinical disease, while a few highly virulent clones are responsible for the bulk of infections worldwide [8].

The age-related curve of *K. kingae* disease closely follows that of oropharyngeal carriage, and >95% of all infections occur before 48 months [1]. Invasive *K. kingae* strains may penetrate the pharyngeal epithelial surface, enter the bloodstream, cause bacteremia, and translocate to distal sites, particularly bones, joints, and the endocardium [1, 6]. Breaching of the pharyngeal mucosal layer by colonizing *K. kingae* organisms is frequently assisted by a recent or concomitant viral infection of the mouth or upper respiratory tract [1].

The clinical presentation of *K. kingae* bacteremia and skeletal system infections is remarkably subtle; patients are in good general condition, many are afebrile, and the blood leukocyte count and acute phase reactant concentrations may be within normal values or show an unimpressive elevation [1, 3]. Because of the subtle presentation of *K. kingae* osteomyelitis, septic arthritis, and spondylodiscitis, and the difficulty in recovering the organism in culture, many cases are diagnosed with considerable delay or missed altogether [1]. However, the overall prognosis of *K. kingae* bacteremia and bone, joint, and intervertebral disc infections is favorable; if adequately treated, patients recover without long-term sequelae [1].

Contrary to the benign features of skeletal system infections, *K. kingae* endocarditis is an aggressive illness characterized by high fever, marked leukocytosis, and elevated inflammation markers [1]. Despite the remarkable susceptibility of the bacterium to antibiotics, the disease progresses relentlessly, causing extensive destruction of valvular tissues [9]. Life-threatening cardiac complications, embolic phenomena, cerebrovascular accidents, and meningitis are common [1]. The mortality rate is high (10%), and many survivors will require surgical correction of the damaged cardiac valves [1, 10].

Despite the substantial oropharyngeal prevalence of *K. kingae* among preschoolers, the annual risk of colonized children developing an invasive infection is low (<1%). Traditionally, the disease cases have been sporadic [11]. In 2005, Kiang et al. reported the first cluster of invasive *K. kingae* infections affecting 3 attendees of a Minneapolis DCC facility [12]. Over the following years, many additional DCC outbreaks have been detected, indicating the emergence of a novel public health threat [13–23]. This review aims to summarize and analyze the outbreaks’ demographic and clinical features to facilitate their early recognition, improve their investigation and management, and prevent potential complications.

## METHODS

*Kingella kingae* outbreaks were defined as the occurrence of ≥2 proven cases (by bacteriological culture and/or nucleic acid amplification tests) of invasive *K. kingae* infections among attendees to a DCC facility within a 40-day period.

The Pubmed database was employed to search for reports of *K. kingae* disease outbreaks in DCC facilities published in English, French, and Spanish. The keywords (“*Kingella kingae*”) AND (“disease outbreaks” OR “disease clusters” OR “daycare centres” OR “daycare facilities” OR “childcare”) were used in the search. The bibliographic research ended on July 31, 2024. Overall, a total of 12 primary source articles resulted from the search. In addition, unpublished and well-documented personal communications known to the author of the present article were also included in the data analysis.

## RESULTS

Overall, 27 DCC outbreaks of invasive *K. kingae* disease were identified; 24 have already been published, and 3 were from personal communication with R. Free (outbreak number 24), N. El Houmami (outbreak number 25), and E. Goyer (outbreak number 27) (Table 1). All these events have been detected in industrialized countries, including the United States (n = 5), Canada (n = 1), Spain (n = 2), France (n = 11), Luxembourg (n = 2), and Israel (n = 6).

**Table 1.** Summary of Clinical and Demographic Features of the 27 Outbreaks of Invasive *K. kingae* Infections in DCCs

Outbreak	Year	Country	Location	No. of Cases	Carriage Rate		MLST		Antibiotic Prophylaxis	References
					No./Total	%	ST	STC		
1	2003	USA	Minneapolis, MN	3	9/17	52.9	23	23/25	+	12
2	2005	Israel	Military base A	3	4/11	36.4	6	6	+	13
3	2007	USA	Durham, NC	3	NA	NA	14	14	+	14
4	2011	France	Paris	5	11/13	84.6	25	23/25	+	15
5	2012	Israel	Eilat	2	4/34	11.8	6	6	+	16
6	2013	France	Marseille	5	14/15	93.3	25	23/25	+	17
7	2013	Israel	Uum-el-Fahm	2	5/11	45.5	6	6	+	16
8	2013	France	Ile-de-France	3	NA	NA	NA	NA	−	18
9	2014	Israel	Military base B	2	4/10	40.0	NA	14	+	19
10	2014	Israel	Kibbutz Nir-Itzhak	2	7/10	70.0	6	6	+	19
11	2014	France	Loire Atlantique	3	NA	NA	NA	NA	−	18
12	2014	Israel	Military base C	2	7/12	58.3	16	14	+	19
13	2014	France	Grand Est	3	NA	NA	NA	NA	−	18
14	2015	Spain	Roses	3	8/9	88.9	25	23/25	+	20
15	2016	France	Nice	2	NA	NA	66	32	+	21
16	2016	France	Châteauneuf-les-Martigues	2	NA	NA	NA	NA	−	18
17	2016	Spain	Madrid	2	NA	NA	NA	NA	−	18
18	2016	France	Pays-de-Loire	2	NA	NA	NA	NA	−	18
19	2016	France	Hauts-de-France	4	NA	NA	NA	NA	−	18
20	2017	USA	New York, NY	2	NA	NA	NA	NA	+	18
21	2017	USA	Valley City, ND	2	NA	NA	NA	NA	+	22
22	2017	France	Ile-de-France	3	NA	NA	NA	NA	−	18
23	2018	Luxembourg	Luxembourg	2	NA	NA	NA	NA	−	18
24	2018	USA	Nebraska	2	NA	NA	NA	NA	NA	PC
25	2018	Luxembourg	Luxembourg City	2	NA	NA	NA	NA	NA	PC
26	2019	France	Amiens	4	NA	NA	25	23/25	−	23
27	2022	Canada	Laurentians	2	NA	NA	NA	NA	+	PC
Total				72	73/142	51.4			15/25	

Abbreviations: DCCs, daycare centers; NA, not available; PC, personal communication; ST, sequence type; STC, sequence type complex.

The patients’ age was known in 60 of the 72 (83.3%) affected attendees, with 57 (95.0%) aged 6–24 months; only 3 were older. The median age of the affected attendees was 14 months, with a mean ± SD of 14.4 ± 4.8 months and a range between 6 and 32 months. The patients’ gender was only reported in 16 outbreaks comprising 43 of the 72 (59.7%) children; 25 (58.1%) were males, and 18 were females. The attack rate in the affected classrooms had a median of 15.5%, a mean ± SD of 17.9% ± 7.1%, and a range from 5.6% to 33.3%. The outbreak duration, calculated as the time lapsed between the onset of symptoms in the first and last affected attendees, had a median of 10.5 days, a mean ± SD of 12.9 ± 9.9 days, and a range of 2–37 days.

The clinical presentation of the affected attendees included the typical spectrum of *K. kingae* disease. Most children (n = 66, 91.7%) had skeletal system involvement, and 3 (4.6%) patients each had bacteremia or endocarditis. Septic arthritis was diagnosed in 31 of the 72 (43.0%) children, osteomyelitis in 26 (36.1%), tenosynovitis or cellulitis in 4 (5.6%), spondylodiscitis in 3 (4.2%), and myositis in 1 (1.4%). The use of magnetic resonance imaging (MRI) technology revealed multiple foci of infection in 6 of the 66 (9.1%) patients with skeletal system disease: combined septic arthritis and osteomyelitis in 2 children (in outbreaks 1 and 6), involvement of 2 joints in 1 child (in outbreak 1), osteomyelitis of 2 bones and 1 focus of arthritis in a single child (in outbreak 23), osteomyelitis of 3 bones in 1 child (in outbreak 23), and tenosynovitis and osteomyelitis in 1 child (in outbreak 25).

All children with skeletal system infections, cellulitis, or bacteremia made an uneventful and full recovery after receiving adequate antibiotic therapy. In contrast, the 3 DCC attendees with endocarditis had a stormy and complicated clinical course. One child developed multiple emboli to the kidney and brain and mitral valve perforation requiring emergency surgical repair (outbreak 15), 1 child had mitral valve insufficiency (in outbreak 3), 2 developed meningitis and cerebral infarctions (in outbreaks 3 and 15), and 2 attendees died (in outbreaks 3 and 27). In 1 of the fatal cases, endocarditis was only diagnosed postmortem (in outbreak 27).

In recent years, detection of the elusive *K. kingae* has been improved by the use of more rapid and sensitive NAATs. Therefore, in 14 of 27 (48.1%) outbreaks, laboratory diagnosis was performed by species-specific NAATs, while in 2 others the isolate was unavailable for genomic analysis. In the remaining 13 outbreaks, the causative strain was recovered in culture and subjected to multilocus sequence typing (MLST).

In 12 (92.3%), the strain belonged to the highly invasive sequence type complexes (STCs) 23/25 (n = 5), 6 (n = 4), and 14 (n = 3), and 1 belonged to the uncommon sequence type (ST) 32 (Table 1). With a few exceptions, pharyngeal isolates detected among healthy carriers during the outbreak investigation were genotypically indistinguishable from the patients’ invasive isolates.

In the 15 of 25 (60%) clusters for which the data were available—including the 3 outbreaks in which a case of endocarditis occurred—prophylactic antibiotics (oral amoxicillin and/or rifampin for 2 or 4 days) were provided, and no further infections were detected in the facility. Additional preventive measures comprised enforcement of hand washing, cleaning of toys, equipment, and surfaces, and temporary closing of the facilities [13–23].

## DISCUSSION

Notably, all 27 clusters of invasive *K. kingae* disease identified so far have been detected in the industrialized world. This finding reflects the large number of young children currently attending DCC facilities in Western countries where many mothers are employed outside of their homes [24, 25]. This social phenomenon has a significant public health effect because the transmission of human pathogens is substantially enhanced in the DCC setting, resulting in increased morbidity and clusters of bacterial and viral infections [24, 25].

It is also remarkable that 11 of the 27 (40.7%) outbreaks were detected by researchers already involved in the study of *K. kingae* and its infections (outbreaks 2, 4–7, 10, 12, 15, 23, and 25). The Luxembourg experience further illustrates the importance of awareness in detecting outbreaks of this elusive organism. Until 2017, no *K. kingae* outbreak had been diagnosed in the country. At the beginning of 2018, Nawal El Houmami, a French pediatric surgeon and a prominent researcher of *K. kingae* disease, moved to Luxembourg City to work at the Kannerklinik [26]. In March 2018, shortly after her arrival, she detected the first cluster of infections in a local childcare facility (outbreak number 23), and a second was detected in October of the same year (outbreak number 25).

The peculiar geographic distribution of the outbreaks overlaps with the sporadic *K. kingae* infections reported in the medical literature, whereas notifications from the developing world are rare [1]. This paucity of reports does not prove that the organism is restricted to Western countries. Instead, it is probably caused by the unavailability of the costly automated blood culture systems and NAATs required to detect this fastidious microorganism in resource-limited countries. However, the worldwide adoption of molecular diagnostic methods in recent years and the increasing acquaintance of clinical microbiology laboratories with *K. kingae*'s microbiological features have resulted in a gradual increase in the number of case reports from South America, Africa, and Oceania, indicating that the bacterium has a universal distribution [27–29].

Many DCC outbreaks follow upper respiratory tract infections, especially hand-foot-and-mouth and hepangina [1, 17, 18, 21, 23]. This association suggests that injury to the pharyngeal epithelium induced by the intercurrent viral disease facilitates bloodstream invasion by colonizing *K. kingae* strains and dissemination to distant sites [1].

In general terms, the outbreaks exhibited similar demographic and clinical features disregarding time and place, and knowledge of these idiosyncratic characteristics may enable timely recognition of the events, guide the conduct of thorough epidemiological investigations, and facilitate early institution of prophylactic measures to contain outbreaks and prevent additional morbidity. The diseased attendees showed a distinctive age distribution of sporadic *K. kingae* infections; almost all children were 6–24 months, and no affected child was older than 32 months. As expected, the skeletal system was involved in the vast majority of affected attendees. In contrast, bacteremia with no focal disease, the second most common manifestation of invasive *K. kingae* infections, was underrepresented in this series [1]. It is possible that additional children were bacteremic, but blood cultures were not obtained because of their good general condition, absence of fever, and lack of leukocytosis [1].

Within each outbreak, the clinical syndromes among attendees at a given facility showed a statistically significant trend to be concordant, suggesting that some *K. kingae* strains had the propensity to invade particular body tissues [30]. For instance, although tenosynovitis is a rare presentation of *K. kingae* disease, 3 of the 5 children involved in the 2013 Île-de-France cluster (outbreak number 8) had a tendon sheath infection [18].

However, it should be pointed out that the proclivity of a given organism to invade the same skeletal structures in multiple hosts is only relative, and many strains conserve the capability to colonize a diversity of tissues and body sites. This competence was evident in the 2007 North Carolina outbreak (outbreak number 3), in which 1 child had spondylodiscitis, another cellulitis, and a third endocarditis and meningitis [14]. This heterogeneous clinical presentation could make it challenging to consider a possible common ground, recognize the outbreak, and institute preventive measures. The occurrence of secondary cases several weeks apart may also blur the outbreak’s identification. Table 2 summarizes the major hurdles in identifying *K. kingae* outbreaks in DCCs.

**Table 2.** Potential Contributors to the Failure to Recognize Clusters of *K. kingae* Infections in DCC Facilities

Potential Contributor	Reason
Failure to suspect a skeletal system infection	Mild clinical presentation; normal blood WBC counts and acute-phase reactant levels
Failure to suspect occult bacteremia and obtain blood cultures	Lack of fever and/or leukocytosis (<15 000 WBC/mm^3^)
Noninformative joint tap	Negative gram stain and, usually, <50 000 WBC/mm^3^ of synovial fluid
Lack of use of blood culture vials for seeding skeletal system exudates	Unawareness
Misidentification of the isolate	Unfamiliarity with *K. kingae*'s microbiological and biochemical features
Lack of use of species-specific NAATs for skeletal system exudates	Unawareness, unavailability
Failure to search for *K. kingae* in pharyngeal specimens	Failure to use selective culture media and species-specific molecular tests
Heterogeneous clinical presentation among DCC attendees	Mixture of skeletal system infections, bacteremia, and/or endocarditis
Cases separated by several weeks	Prolonged and slow-progressing clusters

Abbreviations: DCC, daycare center; NAATs, nucleic acid amplification tests; WBC, white blood cell.

Overall, joint and bone infections are sporadic and relatively uncommon in pediatric practice, and their estimated annual incidence in Western countries ranges between 2 and 10 cases per 100 000 [31–33]. Thus, diagnosing multiple cases of skeletal system infections in the same DCC facility within a short period is highly unusual and should raise immediate suspicion of a *K. kingae* outbreak.

The present report shows that not all children with *K. kingae* disease will suffer a benign infection, and 3 of the 72 sick attendees developed complicated endocarditis, resulting in 2 fatalities. The severe clinical presentation, clinical course, and reserved prognosis of endocardial infection contrast with the benign features of bacteremia and osteoarthritis, suggesting that *K. kingae* endocarditis could be caused by a subpopulation of organisms with increased virulence and tropism toward the cardiac tissues. In a recent study, the genomic content of 18 *K. kingae* isolates from endocarditis patients identified 13 different MLST sequence types, indicating that the capability to invade the heart is widespread among *K. kingae* strains [34]. However, 8 (44%) isolates belonged to sequence type complex (STC) 23/25, which has been previously identified in 2 French patients with endocarditis, suggesting that this highly invasive STc has the propensity to invade the heart [34]. Significant genomic differences were found between *K. kingae* strains isolated from adult and pediatric patients with bacterial endocarditis, asymptomatic pharyngeal carriers, and children with bacteremia and osteoarthritis, suggesting the presence of syndrome-specific genomic determinants [34]. The study could not identify genes whose presence or absence was exclusively associated with bacterial endocarditis. However, disregarding the MLST grouping, several genes were overrepresented among the 18 endocarditis-causing isolates and lacking in nonendocarditis invasive organisms, suggesting that the cardiac tropism exhibited by some strains depends on arrangements of multiple virulence-encoding genes that are distributed throughout the genome [34]. However, it can also be argued that the severe clinical course of patients with *K. kingae* endocarditis does not exclusively result from the biological characteristics of the strain but also from the peculiar microenvironment of cardiac valve vegetations, which is difficult to access for antibiotics, leukocytes, and antibodies [35].

The fatal outcomes of the 2 attendees could support the routine use of prophylactic antibiotics in the affected DCC classrooms to prevent additional cases and avoid severe morbidity. However, there is no universal agreement on the need to administer prophylactic antimicrobial therapy to asymptomatic attendees when single or multiple cases of invasive *K. kingae* disease have been diagnosed in a DCC. The fact that no additional disease cases were detected after administering antibiotic coverage to asymptomatic contacts attending 15 of the affected facilities could suggest effective protection. However, in the remaining 10 DCCs where no prophylaxis was prescribed, the outbreak ended without pharmacological intervention, possibly because of the acquisition of herd immunity induced by the prolonged carriage of the causative strain, extinction of the viral trigger, or both.

The question of the need to prescribe antibiotics to asymptomatic children attending DCCs where clusters are detected remains open, although prophylaxis should probably be administered to those at increased risk of developinging a serious infection such as those with congenital heart disease or immunocompromise. The question for the general population can only be properly addressed in a large prospective, randomized study. However, because of the infrequency of these events, a definitive answer will not be available in the foreseeable future. Meanwhile, it will probably be difficult, if not impossible, to withhold antibiotic prophylaxis from children attending DCCs in which a case of life-threatening endocarditis has occurred.

## CONCLUSIONS

The introduction of a virulent *K. kingae* strain in a DCC attended by young, immunologically naive children may result in its rapid dissemination, causing invasive disease in multiple attendees. These infections may be underrecognized because of their subtle and atypical presentation and the difficulty of isolating the organism. The occurrence of multiple cases of skeletal system infections in a DCC facility within a short period should raise suspicion of a *K. kingae* outbreak. Increased awareness of this emerging public health problem and using sensitive NAATs for detecting infected children and carriers are recommended to identify and curtail outbreaks and prevent fatal outcomes.
